# The influence of social cues in learning and decision-making over time

**DOI:** 10.3389/fpsyg.2026.1691925

**Published:** 2026-07-31

**Authors:** Eliany Perez, Alayna Shoenfelt, Ian Frazier, Dalia El-Shafie, Tian Lin, Nichole R. Lighthall, Natalie C. Ebner

**Affiliations:** 1 Department of Psychology, University of Florida, Gainesville, FL, United States; 2 Department of Psychology, University of Central Florida, Orlando, FL, United States; 3 Center for Cognitive Aging and Memory, McKnight Brain Institute, University of Florida, Gainesville, FL, United States; 4 Institute on Aging, University of Florida, Gainesville, FL, United States

**Keywords:** congruence, faces, Iowa gambling task, learning, social decision-making, uncertainty

## Abstract

**Introduction:**

Initial visual impressions guide judgment and decision-making in social contexts, and social cues, such as facial trustworthiness, may bias choices by influencing individuals to prioritize appearance over actual behavioral outcomes. Key challenges for research in this field include clarifying the extent to which decision-making and learning under uncertainty are affected (1) by facial cues of trustworthiness vs. untrustworthiness, (2) by the congruence vs. incongruence of face-based visual cues and behavioral outcomes, and (3) in a social relative to a non-social decision context.

**Methods:**

This study used the Social Iowa Gambling Task (S-IGT), an adaptation of the Iowa Gambling Task (IGT). Extending traditional decision-making paradigms by systematically integrating several novel aspects, the S-IGT features: (1) the experimental manipulation of facial cues perceived as trustworthy vs. untrustworthy and (2) the incorporation of decision options paired with congruent (CS-IGT) vs. incongruent (IS-IGT) social cues; and was used here in combination with (3) a non-social/neutral control condition (the standard IGT).

**Results:**

Across 558 participants, we found that initial performance in the CS-IGT was higher than in the other two task conditions, yet participants’ learning progress diminished over time in the CS-IGT.

**Discussion:**

These findings suggest that while social cues of facial trustworthiness may support early decision-making, they also may encourage reliance on initial impressions over feedback-driven exploration, thus crucially influencing choice and learning trajectories under uncertainty in social contexts. The results further highlight the complex role of visual facial and behavior-based information congruence vs. incongruence in dynamic social decision environments.

## Introduction

Imagine deciding whether to confide in someone and build a meaningful connection based solely on the fleeting expression on their face—would you trust them enough to share something personal or invest in that relationship? Such minor and automatic evaluations reflect the complexity of real-world behavior, where decision-making is often driven by subtle, non-verbal social cues, especially in contexts where uncertainty or incomplete information creates ambiguity (Eskenazi et al., 2016; Suzuki et al., 2019). In social contexts, these cues often serve as heuristics that help individuals make rapid judgments about the credibility and reliability of others (Jaeger et al., 2019). These heuristics may also be particularly vital in online and in-person interactions where information about another person is limited and where making prompt assessments of familiarity, positivity, or trustworthiness is critical to decision-making (Liu and Goodhue, 2012). That is, as quick, automatic indicators, social cues can instantly influence an individual’s willingness to engage in initial interaction or initiate contact with another person (Van der Biest et al., 2025). However, while these social cues may guide initial social decisions, less is known about how reliance on social cues influences longer-term learning and adaptation, particularly when such cues are incongruent with an individual’s actual behavior and reliability.

Traditional behavioral economic paradigms, like the Trust Game and the Ultimatum Game, have been widely used to examine dynamics pertaining to how social contexts and expectations of fairness and reciprocity influence decisions (Blount, 1995; Frazier et al., 2021; Von Neumann and Morgenstern, 1947). While valuable, these paradigms typically involve direct interpersonal interactions with contingent outcomes, which differ from many real-world scenarios where social information is available but direct reciprocity is absent (Axelrod and Hamilton, 1981; Rilling and Sanfey, 2011). Also, in social contexts, repeated exposures to social cues, such as observing others’ facial expressions, body language, or other visual cues across repeated interactions, shape judgments and influence decisions over and above behavior about whom to trust, confide in, or build relationships with. Facial trustworthiness cues in particular have been shown to capture attention and influence credibility assessments (Bente et al., 2012; Fung et al., 2023; Song and Luximon, 2021). However, the mechanisms through which such face-based social cues impact decision learning, especially under uncertainty, remain less well understood. While social cues may generally support learning and decision-making, it is also possible that salient social information facilitates early choices but then impedes ongoing adaptation; because when this type of powerful social cue is available, individuals may rely less on feedback from behavioral outcomes and more on their initial visual impressions.

Recent theoretical work highlights the role of the explore–exploit trade-off in dynamic decision-making under uncertainty (Perez et al., 2025; Wilson et al., 2014). According to this framework, individuals must balance exploiting options that appear initially advantageous with exploring alternative options that could yield better long-term outcomes. Risk tolerance, learning rates, and optimism about unfamiliar options contribute to these decision strategies. Applied to the present study, reliance on facial trustworthiness cues may reflect an early exploitation of socially salient information, potentially at the expense of broader exploration and adaptive learning based on objective outcomes. The evolving process of trust-building is shaped by implicit learning, or the unconscious acquisition of knowledge through repeated exposure to patterns or cues (Gordon and Holyoak, 1983). In social contexts, implicit learning occurs as individuals encounter repeated interactions, shaping their preferences and biases unconsciously (Yoo, 2008). Subtle social signals, including facial expressions, can thereby exert significant influence on decision processes over time (Eckel and Wilson, 2003; Morris et al., 1998; Willis and Todorov, 2006). These processes are influenced by underlying schemas, or mental frameworks developed through experience, that guide attention, memory, and decision-making (i.e., based on “schemata”; Bartlett, 1932). However, while schemas can support decision-making, they also can introduce biases (Ebner et al., 2009; Lempert et al., 2022; Mather and Johnson, 2003; Owens et al., 1979) and influence behavior (Banting et al., 2009; Lee et al., 2011).

The literature points to an intriguing pattern: facial cues, whether emotional or neutral, when congruent with interaction outcomes, tend to amplify trust or distrust judgments (Campellone and Kring, 2013; Chang et al., 2010). However, the specific influence of face-based social cues in decision-making contexts involving complex social interactions is not understood. This study aimed to bridge this gap by examining how facial cues congruent vs. incongruent with behavioral outcomes affect decision-making strategies, especially when explicit memory retrieval is challenged – an important factor in how individuals evaluate credibility and trustworthiness in dynamic social interactions in real life (Hong et al., 2019; Horta et al., 2024).

### The Social Iowa Gambling Task (S-IGT)

To address these questions, we utilized the Social Iowa Gambling Task (S-IGT; Horta et al., 2024), a recent adaptation of the Iowa Gambling Task (IGT; Bechara et al., 1994) that incorporates social stimuli (facial cues) to explore the impact of perceived trustworthiness on decision-making. The S-IGT features two conditions: (1) a congruent condition (CS-IGT), where trustworthy faces are paired with advantageous decks, and (2) an incongruent condition (IS-IGT), where trustworthy faces are paired with disadvantageous decks. This design, alongside the standard IGT control condition that we also used here, allowed for a nuanced analysis of how social cues influence decision-making in contexts where first impressions and trust play a pivotal role (for a similar approach, see Trotzke et al., 2019).

The IGT’s foundational role in measuring complex decision-making processes and its ecological validity make it an ideal base for adaptation. Previous adaptations, such as the use of spider/butterfly images or emotional facial expressions, have begun to explore the role of external cues in decision-making (Pittig et al., 2014a, 2015). The S-IGT extends this work by focusing on neutral facial expressions with subtle social cues (i.e., facial trustworthiness vs. untrustworthiness) to minimize demand effects and enable direct comparisons between the social (i.e., display of faces in the S-IGT) and non-social (standard IGT) decision-making conditions.

In sum, the S-IGT allows going beyond traditional decision-making paradigms by systematically integrating several novel features, namely (i) the experimental manipulation of facial cues perceived as trustworthy vs. untrustworthy, (ii) the incorporation of decision options paired with congruent (CS-IGT) vs. incongruent (IS-IGT) social cues; and was here used in combination with (iii) a non-social/neutral control condition (the standard IGT).

### Purpose of the current study

The present study leveraged both the recently developed S-IGT and the standard IGT to determine the extent to which decision-making and learning under uncertainty are affected (1) by facial cues of trustworthiness vs. untrustworthiness (by experimentally manipulating the use of trustworthy and untrustworthy-looking faces on card decks in the S-IGT), (2) by congruence vs. incongruence of visual impressions and behavioral outcomes (by experimentally manipulating the pairing of trustworthy/untrustworthy-looking faces with advantageous/disadvantageous card payouts in congruent vs. incongruent ways in the S-IGT), and (3) in a social relative to a non-social decision context (by use of both the S-IGT and the non-social standard IGT).

Specifically, given the influence of social heuristics in social contexts, we anticipated that participants would initially show a preference for choice options paired with trustworthy faces compared to choice options paired with untrustworthy faces in the S-IGT (i.e., first card selection before payout outcome; *Hypothesis 1*). We further predicted that social cues and reward contingencies in the CS-IGT (i.e., trustworthy faces paired with advantageous payouts and untrustworthy faces paired with disadvantageous payouts, relative to the reverse pairing in the IS-IGT) would facilitate learning over time (*Hypothesis 2*). Finally, we followed up on the predictions of Hypothesis 2, by specifically exploring whether enhanced learning in the CS-IGT or impaired learning under the IS-IGT would differ from the non-social standard IGT (*Exploratory Hypothesis 3*).

## Method

### Participants

The university Institutional Review Board approved the study protocol, and all procedures were performed in accordance with relevant ethical guidelines and regulations, including the Declaration of Helsinki. All participants provided written informed consent prior to their study participation.

Participants were recruited from the university undergraduate psychology participant pool (SONA), fliers posted in the community, and word-of-mouth. Phone screenings were conducted to confirm participants were English-speaking and aged 18–35 years. A total of 558 participants were randomly assigned to one of three task conditions: non-social (IGT), congruent-social (CS-IGT), or incongruent-social (IS-IGT; see Table 1 for sample-descriptive statistics and see the Supplementary materials for a sensitivity analysis assessing sample size adequacy).

**Table 1:** Sample description.

Condition	IGT	CS-IGT	IS-IGT
	(*n* = 178)	(*n* = 194)	(*n* = 186)
	*M* (*SD*)	*M* (*SD*)	*M* (*SD*)
Demographics			
Age	21.09 (2.44)	21.24 (2.83)	20.61 (1.68)
% Women	81%	85%	84%
Education	14.32 (1.59)	14.36 (1.61)	14.16 (1.28)

Two participants did not report their gender. Education was measured by the total years of formal education.

### Procedure

Data for this analysis were harmonized across three data collections, that varied in administration mode, incentivization, and stimuli, which enhanced statistical power and broadened generalizability: The first two were conducted in an in-person setting lasting approximately 60 min, with participants receiving monetary compensation (plus a task bonus of up to $10) and the S-IGT used images of middle-aged male faces. The third was an online data collection using the Pavlovia platform, with participants receiving SONA credits (and no task bonus, i.e., absence of a task incentive) and the S-IGT used images of middle-aged female faces.

After written informed consent, participants completed a demographics questionnaire, followed by their assigned task condition (see details below) and socioemotional questionnaires (data not presented here). The session closed with participant debriefing and compensation.

### Standard and Social Iowa Gambling Task conditions

To mitigate potential practice effects observed in previous IGT studies (Bauer et al., 2013; Halfmann et al., 2014; Xiao et al., 2013), we adopted a between-subjects design, with each participant assigned to only one of the three task conditions (IGT, CS-IGT, or IS-IGT). In all conditions, participants started with 2,000 points and were instructed to maximize their points by choosing cards from four decks. Two decks were advantageous, and two were disadvantageous, following a scheme designed to mimic real-life risk/reward scenarios.

#### IGT

The task was programmed in PsychoPy (v3.0.6; Peirce et al., 2019). Each deck of cards in the standard condition was represented by images of Bicycle brand card backs. Consistent with the literature, participants were not aware of the number of times they could draw from the decks or the payout amounts. Participants were told that every draw from a deck would yield some points, but some draws would result in a penalty. Unbeknownst to them, two of the decks were designed to be “disadvantageous” (i.e., resulted in a net loss of 250 points over 10 selections) and the other two to be “advantageous” (i.e., resulted in a net gain of 250 points over 10 selections). That is, cards drawn from disadvantageous decks could result in large rewards but could also result in large punishments. Cards selected from advantageous decks, in contrast, could result in small rewards but could also result in small punishments.

**Figure 1:**
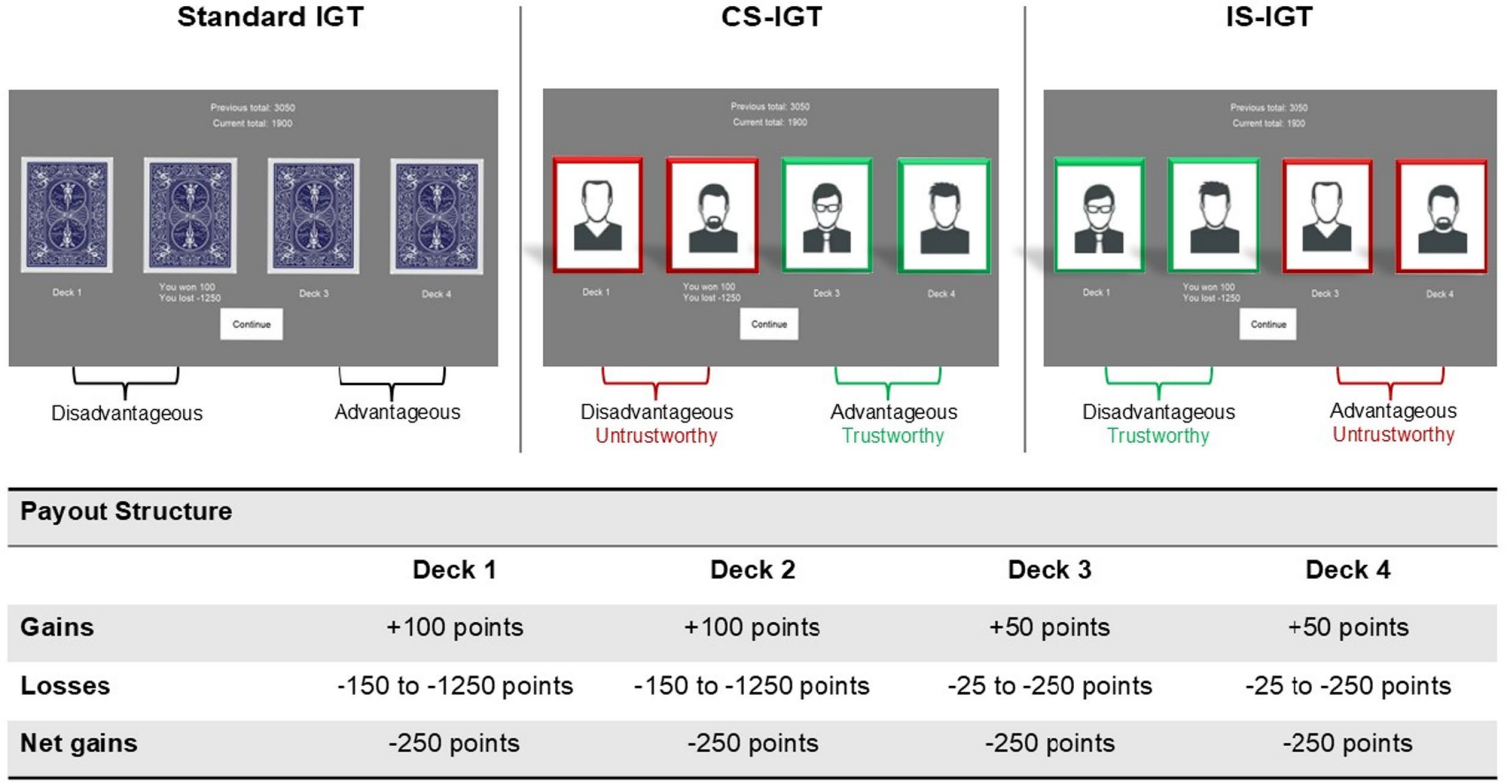
Graphical depiction of the task conditions. The three task conditions in this study were the standard Iowa Gambling Task (IGT), the congruent Social Iowa Gambling Task (CS-IGT), and the incongruent Social Iowa Gambling Task (IS-IGT). In the two social task variants, the decks of cards were represented by face images (not shown here) selected from the FACES Lifespan Database (Ebner et al., 2010) that had been independently rated for perceived trustworthiness (Pehlivanoglu et al., 2023). Advantageous card decks were paired with trustworthy-looking faces (depicted in green) while disadvantageous card decks were paired with untrustworthy-looking faces (depicted in red) in the CS-IGT; this pairing was reversed in the IS-IGT. Participants clicked on a deck to make their selection. Cards drawn from the two decks to the right resulted in immediate small rewards and small delayed punishments (i.e., advantageous decks). Cards drawn from the two decks to the left resulted in large immediate rewards and large delayed punishments (i.e., disadvantageous decks). During the task, results (gains and losses) of a participant’s most recent selection were presented below the selected deck and previous and current totals were updated above the card decks. Participants pressed continue to progress to the next trial.

As shown in Figure 1, during the task, participants were presented with their current and their previous turn’s total points at the top of the computer screen as well as the total number of points they accumulated up to that point in the task. As in the original IGT, both disadvantageous card decks were always located on the left side of the screen and both advantageous card decks always on the right side of the screen (Bechara et al., 1994). All three task conditions followed the reinforcement schedule reported in Bechara et al. (1994) (see Supplementary Table S1 for details).

#### CS-IGT and IS-IGT

In the S-IGT conditions, the instructions from the IGT were modified to frame the task in a social context (see the Supplementary materials for verbatim instructions for all three task conditions). The S-IGT paired faces with each deck and participants were told that previous study participants built the four decks with which they were to play. The face images used to represent each social deck were taken from the FACES Database (Ebner et al., 2010). All faces were of middle-aged adults. As race effects were not targeted in this study, race-associated facial features were not manipulated. All facial expressions were neutral (non-emotional). The four selected face images were the two most and the two least trustworthy-looking faces based on trustworthiness ratings on a scale from 0 (i.e., not trustworthy at all) to 100 (i.e., very trustworthy) obtained from participants in an independent study (Pehlivanoglu et al., 2023; see Supplementary Table S2 for descriptive statistics of the trustworthiness ratings). In the congruent-social condition (CS-IGT), advantageous decks were paired with the two most trustworthy-looking faces and disadvantageous decks with the two least trustworthy-looking faces. In the incongruent-social condition (IS-IGT), this pairing was reversed. We administered two versions of the CS-IGT and two versions of the IS-IGT that switched the face-deck associations within advantageous and disadvantageous decks. For example, in Version 1 of the congruent condition Face 1 was paired with Deck C and Face 2 was paired with Deck D and in Version 2 the faces switched between these two decks. The same was true for the incongruent condition, resulting in five possible conditions – IGT, CS-IGT1, CSIGT2, IS-IGT1, and IS-IGT2. Assignment to these task versions was counterbalanced across participants.

### Statistical analyses

First, to determine if participants were more likely to make their initial selection from a deck represented by a trustworthy compared to an untrustworthy face (*Hypothesis 1*), a single sample Chi-Square analysis on participants’ first selection was conducted across the two S-IGT conditions. Our main analyses examined task performance by 20-trial blocks. Consistent with prior studies (Bauer et al., 2013; Halfmann et al., 2014; Xiao et al., 2013), task performance was determined per block by calculating the total number of draws from advantageous decks minus the total number of draws from disadvantageous decks.

Given the hierarchical data structure (trials nested within participants), we used multilevel modeling (MLM) in Stata 15.1 (StataCorp, 2017. Stata Statistical Software: Release 15. College Station, TX: StataCorp LLC) to test both *Hypothesis 2* that social cues and reward contingencies in the CS-IGT would facilitate learning over time relative to the IS-IGT; as well as *Exploratory Hypothesis 3* that learning over time in the CS-IGT and IS-IGT would differ from the non-social standard IGT. In this model, the outcome variable was task performance with Condition (IGT, CS-IGT, IS-IGT) as a between-subject independent variable and Block (four 20-trial blocks) as a within-subject independent variable. This analysis allowed us to test for a significant two-way interaction between these two factors and to then decompose the predicted interaction using Bonferroni correction for pairwise comparisons for probing our hypotheses under this model. Given gender-imbalance in our sample and aggregation across the three data collections, this model included participant gender (0 = male, 1 = female, 2 = other) and data collection source (0 = Study 1, 1 = Study 2, 2 = Study 3) as covariates.

Importantly, as in the original experiment (Bechara et al., 1994), decks were programmed with only 40 non-replenishing cards, mirroring physical IGT designs. Consequently, some participants ran out of available cards during a potential fifth block. To avoid interpretational biases associated with limited choices, we excluded Block 5 from our primary analyses (for a similar approach, see Horta et al., 2024; results including Block 5 are reported in the Supplementary materials, see Supplementary Table S3 and Supplementary Figure S1).

## Results

Table 2 presents descriptive task performance (advantageous – disadvantageous choices) by Condition and Block. Table 3 summarizes the mean number of deck selections from each deck by Condition and Block. All results reported here reflect analyses conducted on the harmonized dataset (i.e., collapsed across the three data collections and controlled for participant gender and data collection source).

**Table 2:** Task performance: means (standard deviations) [95% confidence intervals].

Block	IGT M (SD) [CI]	CS-IGT M (SD) [CI]	IS-IGT M (SD) [CI]
Block 1	−3.74 (4.21) [−4.36, −3.12]	−2.64 (3.37) [−3.12, −2.16]	−7.12 (3.92) [−7.69, −6.56]
Block 2	−3.08 (4.21) [−3.70, −2.46]	−2.82 (3.37) [−3.30, −2.34]	−4.58 (3.92) [−5.15, −4.02]
Block 3	−0.42 (4.21) [−1.04, 0.21]	−0.19 (3.37) [−0.66, 0.29]	−1.97 (3.92) [−2.54, −1.40]
Block 4	2.80 (4.21) [2.17, 3.42]	0.95 (3.37) [0.47, 1.43]	−0.97 (3.92) [−1.54, −0.40]

Each block comprised 20 card draws. IGT, standard Iowa Gambling Task; CS-IGT, congruent Social Iowa Gambling Task; IS-IGT, incongruent Social Iowa Gambling Task. The reported means refer to the number of choices in the advantageous minus the number of choices in the disadvantageous decks.

**Table 3:** Means and standard deviations of deck selections across blocks and conditions.

Condition	Deck 1	Deck 2	Deck 3	Deck 4
	*M (SD)*	*M (SD)*	*M (SD)*	*M (SD)*
IGT				
Block 1	3.49 (1.78)	8.38 (3.51)	4.06 (3.26)	4.07 (3)
Block 2	3.51 (2.15)	8.03 (3.93)	4.16 (3.39)	4.3 (3.03)
Block 3	2.81 (1.82)	7.39 (4.14)	5.11 (4.32)	4.69 (3.47)
Block 4	2.39 (2.03)	6.21 (4.18)	6.11 (4.67)	5.29 (3.74)
CS-IGT				
Block 1	3.69 (1.38)	7.63 (2.84)	3.79 (2.28)	4.88 (2.95)
Block 2	3.55 (1.94)	7.86 (3.89)	3.96 (2.81)	4.63 (2.98)
Block 3	3.01 (1.65)	7.09 (3.39)	4.37 (2.91)	5.54 (3.05)
Block 4	2.64 (1.79)	6.87 (3.67)	4.59 (2.92)	5.9 (3.25)
IS-IGT				
Block 1	4.25 (1.83)	9.32 (3.08)	2.99 (1.95)	3.44 (1.89)
Block 2	3.69 (1.77)	8.6 (4.01)	3.4 (2.64)	4.31 (2.64)
Block 3	2.95 (1.85)	8.04 (4.04)	4.12 (3.25)	4.9 (3.11)
Block 4	2.84 (1.92)	7.65 (4.4)	4.37 (3.57)	5.14 (3.46)

### Initial deck selection

Consistent with findings from other social decision-making paradigms using facial stimuli (Castle et al., 2012; Chang et al., 2010; Todorov et al., 2009; van’t Wout and Sanfey, 2008; Winston et al., 2002), the single sample Chi-Square for first deck selection in the two S-IGT conditions was significant *χ*^2^(1, 380) = 169.78, *p* < 0.001. First selections were more likely to be from a deck represented by a trustworthy face compared to a non-trustworthy face (317 vs. 63 choices, respectively). Thus, as predicted under *Hypothesis 1*, facial trustworthiness cues biased initial decision-making (toward trustworthy-looking/away from untrustworthy-looking faces) in the absence of information about choice outcomes.

### Choice performance over time

The Condition x Block interaction was significant, Wald *χ*^2^(6) = 24.83, *p* < 0.001. Decomposition of this significant two-way interaction showed that performance over the course of the task was better in the IGT than the CS-IGT, Wald *χ*^2^(3) = 11.22, *p* = 0.01, as well as was better in the IGT than the IS-IGT, Wald *χ*2(3) = 9.49, *p* = 0.02. Further, performance was better in the CS-IGT than the IS-IGT, Wald *χ*^2^(3) = 14.77, *p* = 0.002. As expected, performance in Block 1 was the lowest in the IS-IGT relative to both the CS-IGT (*z* = −7.04, *p* < 0.001) and the IGT (*z* = −4.50, *p* < 0.001), with comparable performance between the CS-IGT and the IGT (*z* = 1.30, *p* = 1.00). By Block 4, however, participants in the IGT outperformed those in the IS-IGT (*z* = 4.08, *p* < 0.001), while performance in the CS-IGT did not significantly differ from that in the IGT (*z* = −2.45, *p* = 0.11) nor the IS-IGT (*z* = 2.03, *p* = 0.34). That is, in contrast to *Hypothesis 2*, participants improved the least in the CS-IGT and the most in the IGT and the IS-IGT – with the latter two task conditions showing overall similar trajectories of improvement by block.

Regarding *Exploratory Hypotheses 3*, we found partial support that the addition of social cues significantly altered learning over time relative to the non-social standard IGT; however, this was in the direction of relatively suppressed learning.

**Figure 2:**
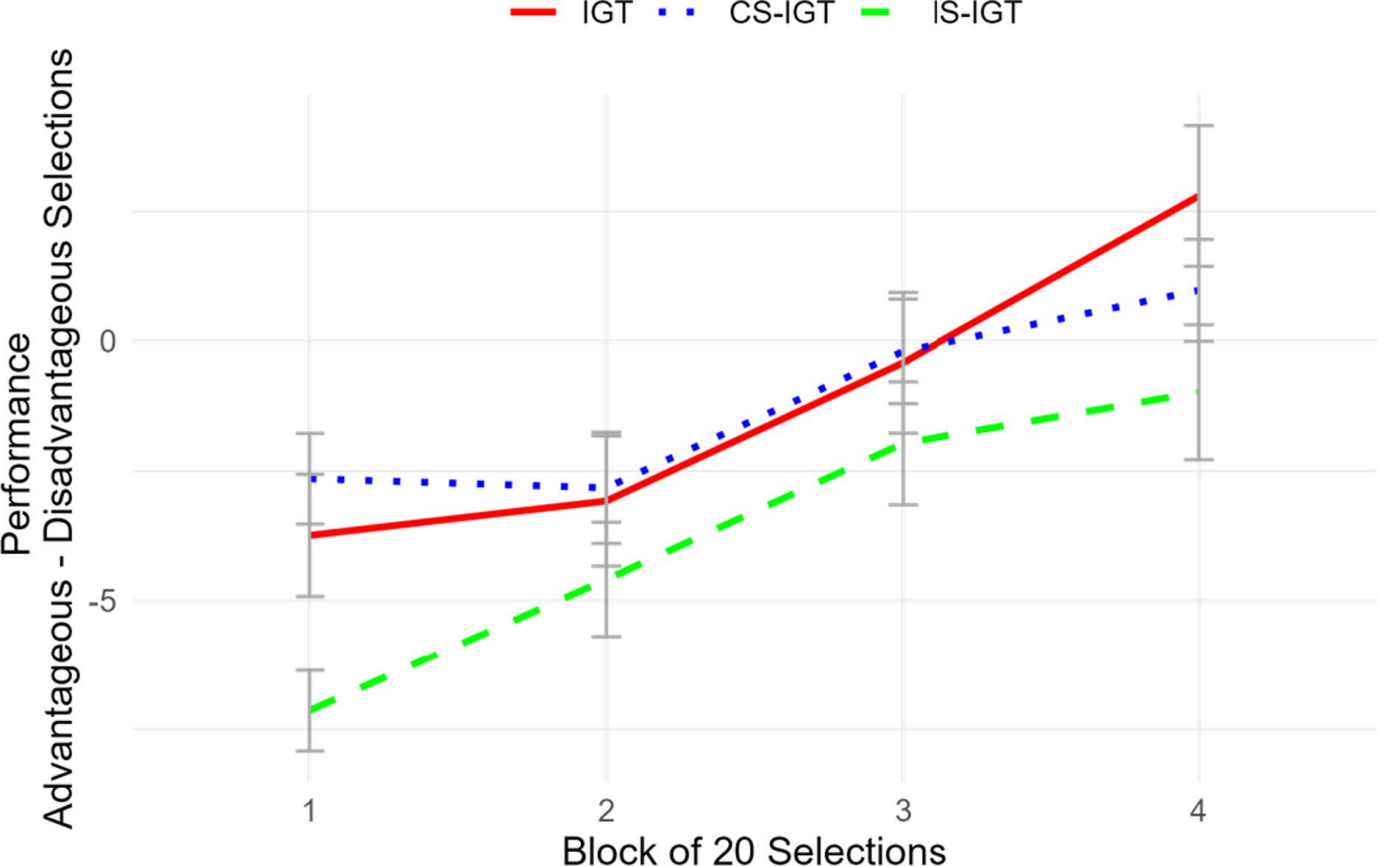
Task performance by task condition: IGT (solid line), congruent (CS-IGT; dotted line), and incongruent (IS-IGT; dashed line). The *x*-axis indicates four blocks of 20 card draws each. The *y*-axis indicates task performance operationalized as the total number of advantageous card draws minus the total number of disadvantageous card draws. Lines depict the raw means and error bars the 95% confidence intervals. The significant Condition X Block interaction (*p* < 0.001) showed that, overall, participants in the IGT outperformed participants in the other conditions (*ps* < 0.01), and performance was better in the CS-IGT than the IS-IGT (*p* = 0.002). Performance in Block 1 was the lowest in the IS-IGT relative to both the IGT and the CS-IGT (*p* < 0.001), while performance was comparable between the CS-IGT and the IGT (*p* = 1.00). By Block 4, participants in the IGT outperformed those in the IS-IGT (*p* < 0.001), and participants in the CS-IGT did not significantly differ from those in the IGT (*p* = 0.129) nor the IS-IGT (*p* = 0.244).

Figure 2 summarizes the task condition effects, which illustrate the impact of facial cues of trustworthiness on initial choice selections as well as subsequent learning trajectories. Notably, participants in the CS-IGT initially benefited from facial cues of trustworthiness, as they made somewhat more advantageous than disadvantageous choices early in the task. However, individuals in this condition showed less improvement in their performance with experience than individuals in either of the other two conditions (IS-IGT and IGT). In contrast, participants in the IS-IGT were initially more hindered by facial cues of trustworthiness but exhibited steep learning trajectories after the initial blocks. Participants in the IGT, which offered neither congruent nor incongruent social information, evidenced greater learning than participants in the CS-IGT and the IS-IGT.

Note that findings regarding our effects of interest (i.e., the selection of trustworthy vs. untrustworthy faces in the analysis on initial deck selection and the Condition x Block interaction in the analysis on choice performance over time) were consistent when analyzing each of the three individual data collections separately as reported in Supplementary Tables S4–S6 as well as when controlling for gender-matching between participant and face stimuli as reported in Supplementary Table S7. Also, while our effects of interest were the same with and without the covariates included, the covariate ‘data collection source’ reached significance (Wald *χ*^2^(2) = 152.50, *p* < 0.001). Pairwise comparisons showed that performance in the online data collection was worse than performance in both in-person data collections (*z*s < 9.70, *p*s < 0.001), but performance between the two in-person data collections did not differ (*z* = 0.11, *p* = 1.00). Thus, while our main results were not affected by this covariate, this finding suggests that in-person vs. online administration constitutes a relevant variable to consider in the use of the S-IGT in future research. Participant gender was not a significant covariate in the model (Wald *χ*^2^(2) = 4.84, *p* = 0.09).

## Discussion

The current study leveraged the Social Iowa Gambling Task (S-IGT), a novel paradigm designed to systematically investigate how trust-related (social) and non-social learning influence decision-making under conditions where visual facial trustworthiness cues are either congruent or incongruent with actual behavioral outcomes (i.e., card payouts; Horta et al., 2024). Our findings reveal significant initial choice performance differences across task conditions, with the CS-IGT condition showing that facial trustworthiness cues aligned with advantageous decks enhanced initial task performance. This effect suggests that social schemas and intrinsic biases play a critical role in early decision-making processes. However, this initial advantage did not extend to long-term learning benefits, presenting an intriguing dynamic between initial impressions and sustained decision-making strategies.

Our results suggest that when trustworthy-looking facial cues are paired with positive outcomes, participants initially defer to these cues, using them as heuristics and thereby perhaps conserving cognitive resources that might otherwise be spent evaluating each choice outcome. This behavior aligns with the concept of satisficing, where individuals opt for a satisfactory rather than optimal solution (Gao et al., 2016). Satisficing may explain why individuals rely on social cues (like endorsements or perceived credibility) to make quick decisions, particularly under time constraints or information overload.

Crucially, however, reliance on facial cues, or social schemas, in both the CS-IGT and IS-IGT conditions appeared to constrain adaptive learning relative to performance in the non-social IGT. This finding suggests that initial impressions derived from social information can exert a persistent influence on decision-making, extending beyond early choice preferences to shape learning trajectories over time. Rather than serving as beneficial sources of information, facial cues may anchor expectations about individuals and bias the interpretation and updating of reinforcement feedback. This interpretation is consistent with research demonstrating that trait inferences are rapidly formed from faces and can influence subsequent judgments and behavior (Todorov et al., 2015). It also aligns with dual-process accounts of social cognition, which propose that automatic evaluations can continue to influence judgments even when more diagnostic information becomes available (Kahneman, 2011), as well as schema-based models of person perception whereby initial trait inferences guide attention and information processing (Fiske and Neuberg, 1990).

Our finding that participants demonstrated superior learning in the non-social IGT compared with both social variants is theoretically significant because it challenges the assumption that social information necessarily facilitates adaptive decision-making. Instead, the presence of social cues may interfere with reinforcement learning processes by introducing competing cognitive demands. From a reinforcement learning perspective, effective performance depends on the accurate integration of reward and punishment feedback across repeated trials (Behrens et al., 2009). Social stimuli, however, are highly salient and may capture attentional resources that would otherwise be allocated to processing outcome information, thereby reducing learning efficiency. This account is consistent with theories of limited cognitive capacity and attentional control (Lavie, 2005), as well as evidence that socially relevant stimuli receive preferential processing. Alternatively, social cues may create expectancy-based biases that alter the weighting of feedback, such that outcomes inconsistent with initial impressions are discounted or integrated less effectively. Future research should directly examine these mechanisms by measuring attentional allocation, cognitive load, and expectancy violation processes during task performance.

Previous studies have paired affective and salient non-social cues with choice options in the IGT and shown that choices align with the valence of the visual cues (butterflies/spiders; Pittig et al., 2014a,b; pleasant/aversive sounds; Priolo et al., 2021). Interestingly, findings in these prior studies for congruent decks are consistent with results in the current study, indicating less learning over time in the congruent than the incongruent condition. However, compared to these earlier studies that did not use face stimuli, in our study overall performance was lower in the incongruent condition due to a strong bias toward decks with trustworthy-looking faces that yielded disadvantageous outcomes over the long run. The overall low performance in the IS-IGT in the current study is comparable to choices made by spider-fearful individuals in Pittig et al. (2014a) when incongruent decks included spider images with advantageous outcomes and were notably avoided by participants. Collectively, these findings across studies indicate that the modulation of visual cues of social trustworthiness has a particularly potent impact on decision-making and learning – especially in cases where one must avoid the “wolf in sheep’s clothing.”

Results of the present study indicated that the CS-IGT was the only condition in which participants, on average, performed better in the first block (i.e., making more advantageous choices) than in subsequent blocks. This is in line with a broad spectrum of research indicating the influence of facial trustworthiness on social decisions (Castle et al., 2012; Chang et al., 2010; Todorov et al., 2009; van’t Wout and Sanfey, 2008; Winston et al., 2002) and aligns with the concept of schema reliance (Ebner et al., 2009; Lempert et al., 2022; Mather and Johnson, 2003). Choosing a trustworthy-looking face in the congruent condition of the S-IGT always resulted in profits and little to no losses since the trustworthy face was paired with an advantageous deck. This likely provided motivation for participants to choose a trustworthy face over an untrustworthy face in this condition.

Further, our results showed that participants in the CS-IGT frequently selected cards from the advantageous decks (paired with trustworthy faces) while occasionally – but not frequently – exploring disadvantageous decks (paired with untrustworthy faces). This strategy aligns with satisficing behavior (Gigerenzer and Todd, 1999), which requires fewer cognitive resources than integrating choice outcomes over trials and uses evolving estimates of choice values to make decisions. In our context, satisficing would mean participants rely on trustworthy faces as a quick heuristic to guide their choices, thereby conserving cognitive resources that would otherwise be expended in meticulously analyzing the outcomes associated with each deck over multiple trials. Thus, a possible explanation for this behavior is that the frequent selection of trustworthy faces paired with advantageous decks in the CS-IGT could reflect convergence on a “good enough” strategy. If participants associate trustworthy faces with positive outcomes, they may be less attentive to feedback or reluctant to explore other options, believing the congruent cues to be reliable indicators of positive outcomes. This mechanism could explain why, despite initially favoring decks associated with trustworthy faces, participants in the CS-IGT displayed slower strategy adjustment over time. This interpretation is supported by our data, showing that participants did engage with disadvantageous decks in the CS-IGT but at a reduced rate compared to the other conditions. The slower rate of strategy adjustment in the CS-IGT could reflect that while participants start with a satisficing approach, namely relying on the heuristic of trustworthy faces, they gradually begin to integrate feedback from their choices, although less efficiently than in conditions without such congruent cues.

This pattern of behavior resembles the findings observed by Pittig et al. (2014a), where participants faced with pictures of butterflies on advantageous decks and pictures of spiders on disadvantageous decks (“no-conflict” condition) were the only group to exhibit advantageous performance in the first block but did not show significant improvement over the rest of the task. While our study parallels Pittig et al. (2014a,b) in terms of initial advantageous performance in the no-conflict condition, a notable divergence arises in the progression of task performance. Unlike in Pittig et al. (2014a,b), our study introduces an essential comparison with the no-cue condition (the standard IGT). This comparison provides valuable insights into the decision-making process. Specifically, it highlights that in the absence of any visual cues, as in the standard IGT, participants’ choices are guided by different dynamics compared to when they are influenced by incongruent visual cues. The initial influence of incongruent visual cues in our task, and the subsequent need for considerable experience to override these initial biases, contrasts with the more stable performance observed in the standard IGT condition as discussed above. This difference underscores the significant impact of visual cues on initial decision making and the learning curve required to adjust in complex decision-making environments.

Divergence between our findings and other work may also be attributable to the nature of the stimuli and task complexity used. For example, the Trust Game in Chang et al. (2010), involving simpler sequential interactions and outcomes, may have facilitated learning processes differently than our more complex IGT environment with multiple choices and mixed gains and losses. This could explain why congruent facial cues had a more pronounced effect in our study, reflecting the complexities of decision-making in real-world scenarios.

Individual differences among participants and variations in experimental design could have further contributed to differences in results. Given the established construct validity of the IGT among clinical populations with decision-making impairments (Buelow and Suhr, 2009), we suggest that in certain contexts (e.g., among individuals with autism spectrum disorders or frontal lobe damage), congruent facial cues of trustworthiness trigger satisficing during the evaluation and integration of social outcomes. Future research should explore boundary conditions under which congruent visual social cues either facilitate or hinder learning, with particular attention to how these mechanisms operate in the context of individual differences in social-cognitive functioning, reward sensitivity, and reliance on heuristic processing.

### Limitations and future research directions

This study broadens our understanding of the intricacies involved in decision-making across varied contexts, extending beyond non-social interactions to incorporate social cues, i.e., facial trustworthiness, central to decision-making. Some limitations of this study offer important opportunities for future research. First, our sample was predominantly female, which could have impacted the findings given that prior research suggests gender differences in trust (van den Akker et al., 2020) as well as in performance on the IGT (van den Bos et al., 2013); but note that inclusion of participant gender and gender-matching between participant and face stimuli as control variables did not change the results for our effects of interest (see the Supplementary Table S7 for details). Also, we used White middle-aged male (Data Collection 1 and Data Collection 2) and female (Data Collection 3) faces in the SIGT. Thus, the facial stimuli in our study were neither specifically age-, gender-, or race-matched to the participants. An interesting avenue for future work would be to systematically vary these facial features to determine their impact on trust learning and decision-making, also in light of known in-group vs. out-group biases in face processing (Hausinger et al., 2025; Kawakami et al., 2022; Rhodes and Anastasi, 2012).

Harmonizing data across three data collection resulted in a larger sample for enhanced statistical power and broader generalizability of our findings across administration mode (in-person vs. online assessment), incentive structure (monetary compensation plus bonus vs. course credit and no bonus), and stimulus properties (male vs. female faces), adding rigor and reliability. This pooling of the data constitutes a strength of the current design as our effects of interested remained stable across the methodological variations (see the Supplemental materials for details, on the results from the analysis conducted on each of the three data collection sources separately). Future research will be able to extend this work by examining additional task features (e.g., instructions) as well as individual differences (e.g., in participant gender, personality, mood) in their impact on S-IGT performance given their demonstrated role in the IGT and learning dynamics (Balodis et al., 2006; Buelow and Suhr, 2013; Hughes et al., 2015; Zanini et al., 2025).

As this study did not assess response times, strategy reports, or cognitive load indicators, we cannot directly speak to the mechanisms behind lower performance among participants in the online vs. the in-person assessment. The administration mode may only be one among other possible explanations for these performance differences. For example, it is possible that ceiling effects, decreased exploration, or differences in motivation or attention also played a role here (DeRight and Jorgensen, 2015; Hauser and Schwarz, 2016), processes that future research will be able to test.

Additionally, although the IGT is a widely used task, some studies have challenged the original theories regarding its psychological processes, suggesting the involvement of more emotional and implicit mechanisms (Brevers et al., 2013; Dunn et al., 2006; Schiebener et al., 2011). Future studies using the S-IGT should examine additional factors influencing decision making, such as incorporating pictures of people from diverse backgrounds to better represent real-world trust interactions. This adaptation could yield insights into how diverse visual cues influence trust-based decisions and preferences. Furthering a mechanistic understanding of these processes, the S-IGT should also be adapted for fMRI and/or ERP paradigms (Halfmann et al., 2014) to allow disentangling the neural mechanisms underlying social vs. non-social decision-making processes. Such adaptations will be particularly valuable for understanding how these processes influence behavior in various populations, including those with decision-making impairments (Le Berre et al., 2017; Saraiya et al., 2019).

Another factor to consider when interpreting our findings is the possibility that traits like altruism, reciprocity, or competition played a role in participants’ choices as they were told that their decisions would affect the bonus of the individuals depicted in the card cue images. For example, highly competitive participants may have had lower effects of facial trustworthiness or greater learning in the IS-IGT condition as outcomes (points) may have been more important to these individuals. Additionally, participants may have perceived the faces not merely as elements within the task but also as cues from the experimenter. Participants’ initial preference for decks with trustworthy faces in the S-IGT could be interpreted as a response to perceived experimenter cues, suggesting “correct” choices from the experimenter’s perspective. Over time, especially in the CS-IGT, participants may have questioned and adjusted this strategy, as these cues did not consistently indicate deck quality. This finding underscores the importance of considering participant perceptions in experimental design on human choice and highlights the need for additional research on how initial impressions can influence longer-term behavior.

## Conclusion

The present study employed the S-IGT as a new paradigm for investigating unique distinctions between social (e.g., trust) and non-social (domain-general) learning and decision-making. Our findings suggest that congruence between a social partner’s appearance and behavior can lead to accurate initial impressions, likely due to schema reliance. However, these initial congruences may ultimately trigger satisficing behavior, which may alter longer-term learning and performance. Participants performed better overall in the IGT, suggesting that social cues do not aid in IGT performance. It may be the case that relying on social schemas increases the demand on mental load and impairs performance when having to make decisions about social interactions. This pattern parallels social behavior, where initial trust signals—such as reputation, prior information, or early social cues—activate heuristic decision-making processes, leading individuals to prioritize familiarity and perceived credibility over deeper cognitive evaluation. Research on social heuristics suggests that as individuals gain experience with others, they rely more on automatic, experience-based judgments rather than systematic analysis, reinforcing rapid decision-making and reducing cognitive effort (Marsh, 2002; Rand et al., 2014), and sometimes at a cost.

Interestingly, participants in the CS-IGT, despite initially benefiting from congruent trustworthy cues, showed less improvement over time compared to those in the other conditions. Conversely, those in the IS-IGT, initially hindered by incongruent cues, exhibited significant learning over time. This divergence in learning trajectories highlights the complex role of visual face-based trustworthiness cues in decision making, suggesting that their influence extends beyond initial impressions and requires adaptive cognitive strategies over time.

Moving forward, it is crucial to test the replicability of our findings using advanced computational modeling and neuroimaging data to explore the underlying neurocognitive mechanisms of social vs. non-social learning (e.g., neural networks supporting social cognition). The S-IGT also has the potential for application to clinical populations that exhibit deficits in trust-related learning and decision-making such as individuals with Parkinson’s disease (Javor et al., 2015) and studies in healthy aging (Horta et al., 2024).

## Data Availability

The datasets presented in this study can be found in online repositories. The names of the repository/repositories and accession number(s) can be found at: https://osf.io/86fdb.
