# Supplementary material for: The influence of social cues in learning and decision-making over time
Source: Front Psychol. 2026 Jul 31;17:1691925. doi: 10.3389/fpsyg.2026.1691925 (PMC13473424; doi:10.3389/fpsyg.2026.1691925)
Supplement: Supplementary file 1 [file Data_Sheet_1.pdf]

SUPPLEMENTAL MATERIALS

The Influence of Social Cues in Learning and Decision-Making Over Time

Eliany Perez[1+], Alayna Shoenfelt[1+], Ian Frazier[1], Dalia El-Shafie[1], Tian Lin[1], Nichole R. Lighthall[2], & *Natalie C. Ebner[1,3,4]

[1]Department of Psychology, University of Florida, Gainesville, FL, USA

[2]Department of Psychology, University of Central Florida, Orlando, Florida, USA

[3]Center for Cognitive Aging and Memory, McKnight Brain Institute, University of Florida, Gainesville, FL, USA

[4]Institute on Aging, University of Florida, Gainesville, FL, USA

Sensitivity Analysis for Assessing Sample Size Adequacy

This study harmonized data from three data collections. No formal a-priori power analysis was undertaken due to the preliminary nature of our inquiry, but we leaned on past literature for sample size determination. Notably, prior research on decision-making and the IGT showcases a broad range of sample sizes, some of which were small-scale for investigative purposes. Considering the scant literature detailing the influence of social cues on the specific task at-hand, our sample size was inspired by prior work that aligned conceptually (Chang et al., 2010; Pittig et al., 2015). We also conducted a sensitivity analysis to determine the smallest detectable effect size at the specified alpha and power levels given our sample size. The sensitivity analysis was conducted in G*Power 3.1.9.7 (Faul et al., 2009) for a repeated-measures ANOVA as an approximation for the multilevel model applied here given the high similarity between these analyses (see Pehlivanoglu et al., 2023 for a similar approach). We set the alpha to 0.05 and the power to 0.90 for our sample of 558 participants. Specifically, for our hypothesis testing of an interaction between Condition and Block, the sensitivity analysis showed that our sample was sufficiently powered to allow detection of a small effect size (Cohen's $f^2 = 0.06$). Thus, the sensitivity analysis confirmed sufficient statistical power and our ability to detect genuine effects.

Reinforcement Schedule

Table S1

Trial-by-trial breakdown of the reinforcement schedule for all three task versions (IGT, CS-IGT, IS-IGT)

	Disadvantageous Deck A		Disadvantageous Deck B		Advantageous Deck C		Advantageous Deck E	
**Trial**	*Gain*	*Loss*	*Gain*	*Loss*	*Gain*	*Loss*	*Gain*	*Loss*
1	100	0	100	0	50	0	50	0
2	100	0	100	0	50	0	50	0
3	100	-150	100	0	50	-50	50	0
4	100	0	100	0	50	0	50	0
5	100	-300	100	0	50	-50	50	0
6	100	0	100	0	50	0	50	0
7	100	-200	100	0	50	-50	50	0
8	100	0	100	0	50	0	50	0
9	100	-250	100	-1250	50	-50	50	0
10	100	-350	100	0	50	-50	50	-250
11	100	0	100	0	50	0	50	0
12	100	-350	100	0	50	-25	50	0
13	100	0	100	0	50	-75	50	0
14	100	-250	100	-1250	50	0	50	0
15	100	-200	100	0	50	0	50	0
16	100	0	100	0	50	0	50	0
17	100	-300	100	0	50	-25	50	0
18	100	-150	100	0	50	-75	50	0
19	100	0	100	0	50	0	50	0
20	100	0	100	0	50	-50	50	-250
21	100	0	100	-1250	50	0	50	0
22	100	-300	100	0	50	0	50	0
23	100	0	100	0	50	0	50	0
24	100	-350	100	0	50	-50	50	0
25	100	0	100	0	50	-25	50	0
26	100	-200	100	0	50	-50	50	0
27	100	-250	100	0	50	0	50	0
28	100	-150	100	0	50	0	50	0
29	100	0	100	0	50	-75	50	-250
30	100	0	100	0	50	-50	50	0
31	100	-350	100	0	50	0	50	0
32	100	-200	100	-1250	50	0	50	0

33	100	-250	100	0	50	0	50	0
34	100	0	100	0	50	-25	50	0
35	100	0	100	0	50	-25	50	-250
36	100	0	100	0	50	0	50	0
37	100	-150	100	0	50	-75	50	0
38	100	-300	100	0	50	0	50	0
39	100	0	100	0	50	-50	50	0
40	100	0	100	0	50	-75	50	0

Note. This trial-by-trial breakdown of the reinforcement schedule was taken from the standard IGT protocol (Bechara et al., 1994).

Task Instructions

IGT

1. In front of you on the screen, there will be four decks of cards 1, 2, 3, and 4.
2. I want you to select one card at a time, by clicking on the card, from any deck you choose.
3. Each time you select a card from a deck, the computer will tell you that you won some points. I won't tell you how many points you will win. You will find out along the way. Every time you win, the number of points will increase.
4. Every so often, however, when you click on a card, the computer tells you that you won some points, but then it says that you also lost some points. I won't tell you when you will lose or how much you will lose. You will find out along the way. Every time you lose, the number of points will decrease.
5. You are absolutely free to switch from one deck to another any time you wish.
6. The goal of the game is to win as many points as possible and, if you find yourself unable to win, make sure you avoid losing points as much as possible. Your points determine your bonus payout of up to 10 dollars at the end of the session.
7. I won't tell you for how long the game will continue. You must keep on playing until the computer stops.
8. You will get 2000 points to start the game. At the end, we will see how much you won or lost.
9. You will not lose points at random. However, there is no way for you to figure out when you will lose points. All I can say is that you may find yourself losing points on all of the decks, but some decks will make you lose more than others. You can win if you stay away from the worst decks.

Any questions?

CS-IGT and IS-IGT

1. In front of you on the screen, there will be four decks of cards that have been created by four participants who have been in the study with us before you. Each deck is presented along with a photo from an existing face database to protect the participants' identities. These faces have been selected by the participants to represent their decks.
2. I want you to select one card at a time, by clicking on the card, from any deck you choose.
3. Each time you select a card from a deck, the computer will tell you that you won some points. I won't tell you how many points you will win. You will find out along the way. Every time you win, the number of points will increase.
4. Every so often, however, when you click on a card, the computer tells you that you won some points, but then it says that you also lost some points. I won't tell you when you will lose or how much you will lose. You will find out along the way. Every time you lose, the number of points will decrease.
5. You are absolutely free to switch from one deck to another any time you wish.
6. The goal of the game is to win as many points as possible and, if you find yourself unable to win, make sure you avoid losing points as much as possible. Your points determine your bonus payout of up to 10 dollars at the end of the session. Every point you lose will go to a bonus payout for the participant who built that very deck. At the end of the study, you will be asked to build your own deck for future participants to play with.
7. I won't tell you for how long the game will continue. You must keep on playing until the computer stops.
8. You will get 2000 points to start the game. At the end, we will see how much you won or lost.
9. You will not lose points at random. However, there is no way for you to figure out when you will lose points. All I can say is that you may find yourself losing points on all of the decks, but

some decks will make you lose more than others. You can win if you stay away from the worst decks.

Any questions?

Independent Facial Trustworthiness Ratings

Table S2

Means (standard deviations) of facial trustworthiness ratings by an independent sample of male and female raters for the stimuli used in the S-IGT

	**Face 1**	**Face 2**	**Face 3**	**Face 4**
	M (SD)	*M (SD)*	*M (SD)*	*M (SD)*
Male Face	59.70 (22.56)	62.10 (15.54)	48.84 (25.50)	48.91 (28.49)
Female Face	66.52 (20.52)	67.73 (14.59)	45.73 (22.24)	47.18 (19.69)

Note. Male and female faces were rated by an independent sample of N = 136 young and older male and female raters. Ratings were made on a scale from 0 (Not at all trustworthy) to 100 (Extremely trustworthy), with higher ratings indicating greater facial trustworthiness. Full data for these independent trustworthiness ratings are reported in Pehlivanoglu et al. (2023).

Parallel Analyses with Block 5 Data Included

Table S3

Task performance: Means (standard deviations) [95% confidence intervals]

	IGT	CS-IGT	IS-IGT
**Block 1** *M* (*SD)* [*CI*]	-3.74 (4.38) [-4.39, -3.09]	-2.65 (3.25) [-3.11, -2.19]	-7.14 (3.88) [-7.70, -6.58]
**Block 2** *M* (*SD*) [*CI*]	-3.08 (4.38) [-3.73, -2.43]	-2.82 (3.25) [-3.29, -2.36]	-4.59 (3.88) [-5.15, -4.03]
**Block 3** *M* (*SD*) [*CI*]	-0.42 (4.38) [-1.06, 0.23]	-0.20 (3.25) [-0.66, 0.26]	-1.97 (3.88) [-2.53, -1.41]
**Block 4** *M* (*SD*) [*CI*]	2.80 (4.38) [2.15, 3.45]	0.98 (3.25) [0.52, 1.44]	-0.98 (3.89) [-1.55, -0.42]
**Block 5** *M* (*SD*) [*CI*]	2.09 (4.38) [1.44,2.74]	2.56 (3.25) [2.10,3.02]	-0.05 (3.89) [-0.62, 0.51]

Note. Each block comprised 20 card draws. IGT = standard Iowa Gambling Task; CS-IGT = congruent Social Iowa Gambling Task; IS-IGT = incongruent Social Iowa Gambling Task. The reported means refer to the number of choices in the advantageous minus the number of choices in the disadvantageous decks.

Figure S1

Task performance by task condition: standard (IGT; solid line), congruent (CS-IGT; dotted line), and incongruent (IS-IGT; dashed line)

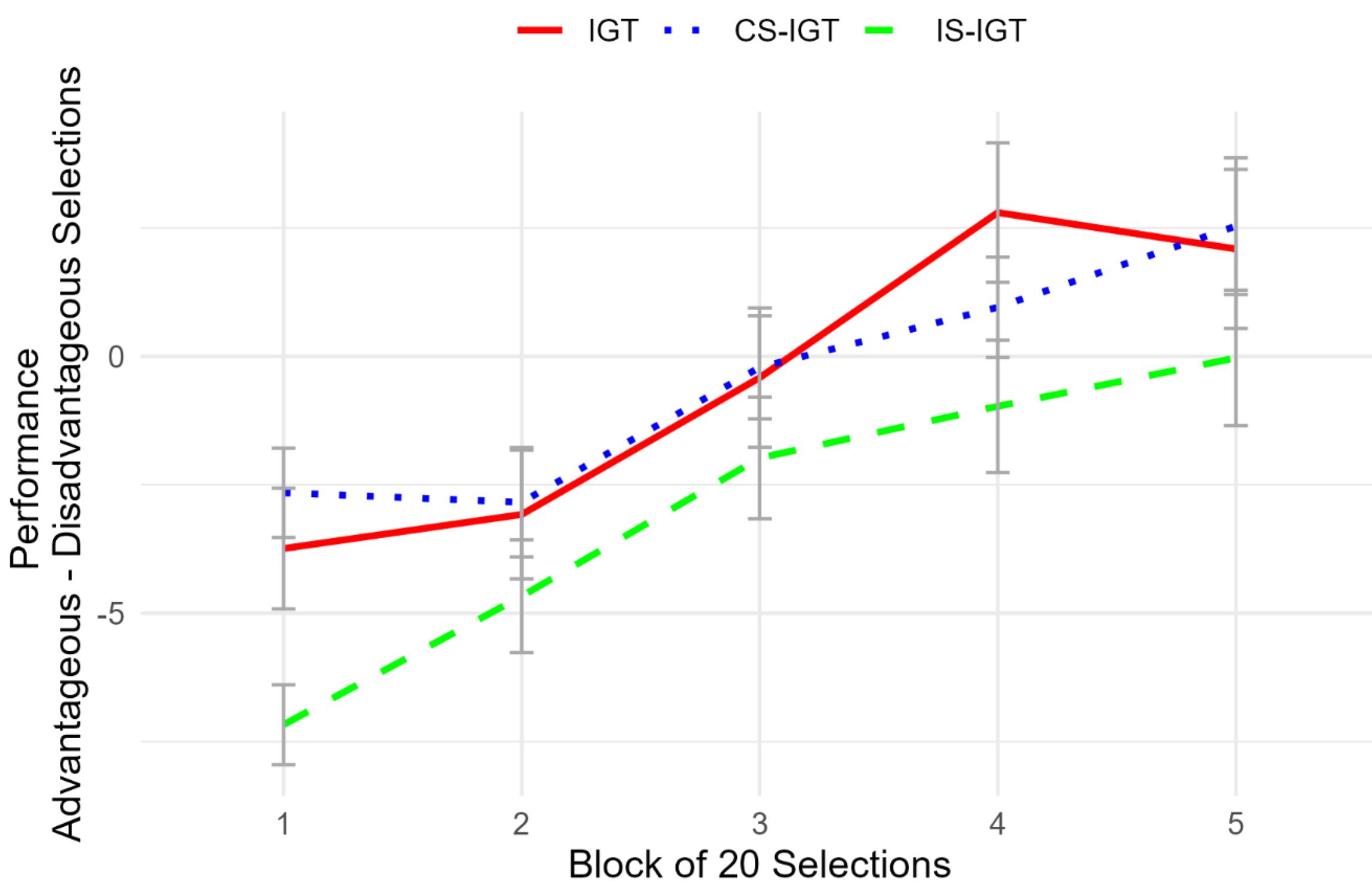

Note. The x-axis indicates four blocks of 20 card draws each. The y-axis indicates task performance operationalized as the total number of advantageous card draws minus the total number of disadvantageous card draws. Lines depict the raw means and error bars the 95% confidence intervals. The significant Condition X Block interaction (p = 0.001) showed that performance was better in the IGT than the IS-IGT (p < 0.001) but not the CS-IGT (p = 0.94), and performance was better in the CS-IGT than the IS-IGT (p < 0.001). Performance in Block 1 was the lowest in the IS-IGT relative to both the IGT and the CS-IGT (ps < 0.001), while performance was comparable between the CS-IGT and the IGT (p = 1.00). By Block 5, participants in the IGT outperformed those in the IS-IGT (p = 0.001), and participants in the CS-IGT did not significantly differ from those in the IGT (p = 1.00) nor the IS-IGT (p = 0.06)

Results By Data Collection Source

For the analysis in this paper, three data collection sources were harmonized. To provide some context, we initially collected data in person in this project across two independent studies (referred to as Data Collection 1 and 2). We then had the opportunity to add to the analysis data we had collected in parallel online, on a modified version of the S-IGT (i.e., use of female faces, the online data collection platform, and absence of monetary compensation and a bonus as incentive as course credits were given to the online participants). This addition of Data Collection 3 allowed us to increase the sample size for greater statistical power and extend the generalizability of the findings given the methodological variations.

Initial Deck Selection

Table S4

Number and inference statistic of initial deck selections by trustworthy/untrustworthy face in each of the three data collections

	Trustworthy Face	Untrustworthy Face	Chi-Square Test
**Data Collection 1 (in-person)**	40	9	$X^2(1,49) = 19.61$, $p < 0.001$
**Data Collection 2 (in-person)**	33	15	$X^2(1,48) = 6.75$, $p = 0.001$
**Data Collection 3 (online)**	244	39	$X^2(1,283) = 148.50$, $p < 0.001$

Choice Performance Over Time

Table S5

Test statistics for effects of interest in each of the three data collections

	Condition	Block	Condition x Block
**Data Collection 1 (in-person)**	$X^2(2) = 4.09$, $p = 0.129$	$X^2(3) = 74.16$, $p < 0.001$	$X^2(6) = 21.57$, $p = 0.002$
**Data Collection 2 (in-person)**	$X^2(2) = 1.80$, $p = 0.406$	$X^2(3) = 34.43$, $p < 0.001$	$X^2(6) = 11.88$, $p = 0.060$
**Data Collection 3 (online)**	$X^2(2) = 32.21$, $p < 0.001$	$X^2(3) = 167.03$, $p < 0.001$	$X^2(6) = 14.73$, $p = 0.020$

Table S6

Comparisons of task performance in task conditions within each block for each of the three data collections

	CS-IGT vs. IGT	IS-IGT vs. IGT	CS-IGT vs. IS-IGT
**Data Collection 1 (in-person)**			
Block 1	$z = 1.39$, $p = 1.000$	$z = -1.09$, $p = 1.000$	$z = 3.57$, $p = 0.003$
Block 2	$z = 0.24$, $p = 1.000$	$z = -0.01$, $p = 1.000$	$z = 0.33$, $p = 1.000$
Block 3	$z = -1.85$, $p = 0.520$	$z = -1.26$, $p = 1.000$	$z = -0.50$, $p = 1.000$
Block 4	$z = -3.30$, $p = 0.010$	$z = -2.20$, $p = 0.220$	$z = -1.04$, $p = 1.000$
**Data Collection 2 (in-person)**			
Block 1	$z = -0.22$, $p = 1.000$	$z = -1.44$, $p = 1.000$	$z = 1.39$, $p = 1.000$
Block 2	$z = 0.29$, $p = 1.000$	$z = 0.44$, $p = 1.000$	$z = -0.17$, $p = 1.000$
Block 3	$z = -0.86$, $p = 1.000$	$z = -0.60$, $p = 1.00$	$z = -0.22$, $p = 1.000$
Block 4	$z = -2.59$, $p = 0.080$	$z = -0.30$, $p = 1.00$	$z = -2.77$, $p = 0.050$
**Data Collection 3 (online)**			
Block 1	$z = 1.44$, $p = 1.000$	$z = -4.56$, $p < 0.001$	$z = 7.11$, $p < 0.001$
Block 2	$z = -0.16$, $p = 1.000$	$z = 2.32$, $p = 0.160$	$z = 2.29$, $p = 0.180$
Block 3	$z = 1.27$, $p = 1.000$	$z = -1.20$, $p = 1.000$	$z = 2.74$, $p = 0.050$
Block 4	$z = -0.73$, $p = 1.000$	$z = -3.91$, $p = 0.001$	$z = 3.61$, $p = 0.002$

Note. Bonferroni-adjusted contrasts are reported.

Moderator Analyses for Gender-Matching Between Participant and Face Stimuli

Given gender-imbalances in our sample distributions and variations of use of male vs. female faces across data collection sources, we conducted follow-up analyses to determine the robustness of our effects of interest (the Condition (CS-IGT vs. IS-IGT) x Block (1 - 4) interaction) when considering gender-matching (0 = no, 1 = yes) as moderator in the analysis. The proportion of gender-matched participants was 44.90% in Data Collection 1, 22.92% in Data Collection 2, and 87.19% in Data Collection 3; and across the full sample, 73.54% of participants were gender-matched.

Table S7 shows results separately for each data collection source as well as across the harmonized sample. Notably, the Condition x Block interaction remained significant when including the moderator, supporting the robustness of our effects of interest. In the harmonized sample, the main effect of the Gender-Matched moderator was significant, in that gender-matched participants performed worse than those who were not gender-matched ($b = -3.57$, $z = -6.40$, $p < 0.001$).

Table S7

Test statistic and p-value for the main effects and interactions of Condition, Block, and (the moderator) Gender-Matched for each of the three data collections separately as well as across the harmonized sample.

	Data Collection 1 (in-person)	Data Collection 2 (in-person)	Data Collection 3 (online)	Full sample
**Condition**	$X^2(1) = 0.59$, $p = .441$	$X^2(1) = 0.03$, $p = .862$	$X^2(1) = 11.94$, $p = .001$	$X^2(1) = 8.61$, $p = .003$
**Block**	$X^2(3) = 47.80$, $p < .001$	$X^2(3) = 26.94$, $p < .001$	$X^2(3) = 49.65$, $p < .001$	$X^2(3) = 115.64$, $p < .001$
**Gender-Matched**	$X^2(1) = 0.73$, $p = .394$	$X^2(1) = 0.20$, $p = .899$	$X^2(1) = 4.39$, $p = .036$	$X^2(1) = 41.00$, $p < .001$
**Condition x Block**	$X^2(3) = 14.54$, $p = .002$	$X^2(3) = 8.57$, $p = .036$	$X^2(3) = 10.77$, $p = .013$	$X^2(3) = 19.85$, $p < .001$
**Condition x Gender-Matched**	$X^2(1) = 0.15$, $p = .697$	$X^2(1) = 0.13$, $p = .717$	$X^2(1) = 0.02$, $p = .885$	$X^2(1) = 4.34$, $p = .037$
**Block x Gender-Matched**	$X^2(3) = 2.05$, $p = .563$	$X^2(3) = 8.59$, $p = .035$	$X^2(3) = 1.45$, $p = .695$	$X^2(3) = 3.68$, $p = .298$
**Condition x Block x Gender-Matched**	$X^2(3) = 1.31$, $p = .728$	$X^2(3) = 1.02$, $p = .797$	$X^2(3) = 4.96$, $p = .174$	$X^2(3) = 7.77$, $p = .051$

Note: Data Collection 1 and 2 used male faces, Data Collection 3 used female faces.
